# Active Transport of Bile Acids Decreases Mucin 2 in Neonatal Ileum: Implications for Development of Necrotizing Enterocolitis

**DOI:** 10.1371/journal.pone.0027191

**Published:** 2011-12-05

**Authors:** Nina A. Martin, Sarah K. Mount Patrick, Teresa E. Estrada, Harrison A. Frisk, Daniel T. Rogan, Bohuslav Dvorak, Melissa D. Halpern

**Affiliations:** Department of Pediatrics and Steele Children's Research Center, University of Arizona, Tucson, Arizona; Wadsworth Center, New York State Dept. Health, United States of America

## Abstract

Necrotizing enterocolitis (NEC) is the most common gastrointestinal emergency of premature infants, but its etiology remains unclear. We have previously shown that mucin 2 (Muc2) positive goblet cells are significantly decreased in NEC. We have also shown that ileal bile acids (BAs) are significantly increased during the development of this disease. Because BAs can affect mucins, we hypothesized that elevated ileal BAs contribute to decreased Muc2 in experimental NEC. The role of Muc2 in NEC was evaluated in Winnie +/+ mice, a strain that produces aberrant Muc2. Muc2 and trefoil factor 3 (Tff3) were assessed in neonatal rats subjected to the NEC protocol when bile acids were removed, and in ileal explants from newborn and older rats cultured with and without BAs. Further, the role of active transport of BAs was determined using neonatal rats given the apical sodium dependent bile acid transporter (Asbt) inhibitor SC-435 and in neonatal Asbt knockout mice subjected to the NEC protocol. Mice with aberrant Muc2 had significantly greater incidence and severity of NEC. Using both *in vivo* and *ex vivo* techniques, we determined that BAs decrease Muc2 positive cells in neonatal but not older ileum. However, Tff3 positive cells are not decreased by BAs. In addition, active transport of BAs is required for BAs to decrease Muc2 in immature ileum. These data show that functional Muc2 plays a critical role in the prevention of NEC and BAs can potentiate the decreased Muc2 in disease development. Further, BAs have a more profound effect on Muc2 in immature versus older ileum, which may explain at least in part why NEC occurs almost exclusively in premature infants.

## Introduction

Despite recent advances in neonatal practice, necrotizing enterocolitis (NEC) remains a major cause of morbidity and mortality in preterm infants [1], [2]. Severe NEC is characterized by an extensive hemorrhagic inflammatory necrosis of the distal ileum and proximal colon [3]. The pathophysiology of this disease remains poorly understood; however, prematurity, enteral feeding, intestinal hypoxia-ischemia, and bacterial colonization are considered major risk factors [4], [5].

Bile acids (BAs) facilitate emulsification, absorption and transport of fats and sterols in the intestine and liver and are essential for normal digestion. However, accumulation of BAs in the intestine has been shown to damage the intestinal epithelium [6]–[8]. Using the neonatal rat model of NEC, we have shown that BAs accumulate in the ileal lumen and enterocytes during disease development. These increased levels of BAs are positively correlated with disease severity. Importantly, when BAs are not allowed to accumulate, neonatal rat pups develop significantly less NEC [9], [10]. In addition, up-regulation of the apical sodium-dependent bile acid transporter (Asbt), which actively transports bile acids from the intestinal lumen into enterocytes, is associated with increased incidence and severity of experimental NEC and is increased in human NEC [11].

Mucins protect the epithelial surface of the GI tract by forming a semi-permeable mucous layer between the lumen and the intestinal epithelium [12], [13]. In premature infants, a deficiency in the mucous layer has been suggested to contribute to intestinal injury during NEC [14]. We have previously reported that ileal mucin 2 (Muc2), the predominant secreted mucin produced by intestinal goblet cells, is significantly decreased in neonatal rats with NEC [15]. Transport of BAs via mucin is a critical step in normal fat absorption and Muc2 has also been shown to be altered by BAs [16]–[19]. Therefore, we hypothesized that BAs could affect ileal Muc2 production, resulting in decreased Muc2 positive cells during the development of neonatal NEC.

The first aim of these studies was to determine if diminished Muc2 exacerbates the development of NEC by subjecting a Muc2 mutant mouse strain to the NEC protocol. In addition, we examined if ileal BAs play a role in the decreased Muc2 positive cells seen in experimental NEC. Using the neonatal rat model of NEC, we examined the effect of BAs on ileal Muc2 positive cells, Muc2 mRNA expression and Muc2 secretion. Rat ileal explant cultures were utilized to determine that neonatal ileum responds differently to BAs than ileum from older rat pups, and that BAs do not universally effect neonatal ileal goblet cell products in the same manner. In addition, ileal explants from Asbt knockout mice and rat ileum treated with the Asbt inhibitor SC-435 were used to determine if active transport of BAs was required to decrease Muc2 in neonatal ileum.

## Materials and Methods

### Animal models

All protocols were approved by the Animal Care and Use Committee of the University of Arizona (A-324801-95081). All animals were monitored for signs of distress during the study (significant abdominal distension, respiratory difficulty, etc.) and sacrificed prior to the end of the study if excessive distress was observed. Tissues from animals sacrificed before the end of the study were included in the analyses.

### Mouse NEC models

Newborn Winnie +/+ and −/− mice (kindly provided by Dr. Michael McGuckin, University of Queensland, South Brisbane, Australia and Dr. Christopher Goodnow, The Australian National University, Canberra, Australia) were collected immediately after birth to prevent suckling of maternal milk and divided into the following experimental groups: **Winnie +/+ NEC** (n = 17) and **Winnie −/− NEC** (n = 10), where pups were hand-fed cow's milk-based formula every 2–3 hours for up to 3 days using the Hoshiba Nipple: Yajima style [20] and dam-fed (DF) **Winnie +/+ DF** (n = 10) and **Winnie −/− DF** (n = 10), where pups were allowed to nurse from a lactating dam. All pups were exposed to asphyxia - N_2_ gas for 30 seconds followed by cold −4°C for 5 minutes - (A/C stress) twice daily [11].

129-Slc10a2 −/− (Asbt KO) and 129 mice (WT) were purchased from The Jackson Laboratory (Bar Harbor, ME). Newborn mice were collected immediately after birth to prevent suckling of maternal milk and divided into the following experimental groups: **Asbt KO NEC** (n = 10) and **WT NEC** (n = 10), where pups were hand-fed cow's milk-based formula every 2–3 hours for up to 3 days using the Hoshiba Nipple: Yajima style and **Asbt DF** (n = 10) and **WT DF** (n = 10), where pups were allowed to nurse from a lactating dam. All pups were exposed to A/C stress twice daily [11].

### Rat NEC models

Sprague-Dawley rats, delivered via caesarian section 1 day prior to scheduled birth, were divided into the following groups: **DF** (n = 12), pups allowed to feed from the mother for 4 days and exposed to A/C stress (N_2_ gas for 60 seconds and 4°C for 10 minutes) [21]–[23]; **NEC** (n = 12), pups hand-fed with formula for 4 days and exposed to A/C stress, **NEC+Chol** (n = 12); pups hand-fed with formula for up to4 days, A/C stressed and given 120 mg/kg/day cholestyramine.

### Disease Evaluation

Pathologic changes in intestinal architecture were evaluated using our previously published NEC scoring system [10], [21], [24]. Histological changes were scored by a blinded evaluator and graded as follows: 0 (normal) – no damage; 1 (mild) - slight submucosal and/or lamina propria separation; 2 (moderate) - moderate separation of submucosa and/or lamina propria, and/or edema in submucosal and muscular layers; 3 (severe) – severe separation of submucosa and/or lamina propria, and/or severe edema in submucosa and muscular layers, region villous sloughing; 4 (necrosis) – loss of villi and necrosis. Intermediate scores of 0.5, 1.5, 2.5 and 3.5 were also utilized to more accurately assess levels of ileal damage when necessary [21]–[26]. To determine the incidence of NEC, only animals with histologic scores of 2 or greater were considered to have developed experimental NEC [21]–[26].

### BA levels

Total ileal luminal BA levels were determined by flushing a section of distal ileum with cold PBS. After flushing, the ileal segment was weighed. Total intra-enterocyte BA levels were determined from the same piece of weighed ileum after homogenization in PBS and centrifugation to separate solid from liquid [10]. BA containing supernatants were frozen at −70°C until assayed and BA levels were determined using the Total Bile Acids Assay Kit (Diazyme, San Diego, CA) according to the manufacturer's protocol.

### Ileal explant culture

Ileal tissue from fasted Sprague-Dawley rats or 129-Slc10a2 −/−(Asbt KO) and wild-type (Asbt WT) was removed and cut into sections. Each section was opened longitudinally on sterile Metricel Membrane Filters (Pall Corporation, Ann Arbor MI) and cultured in DMEM/F12 media alone or with 0. 25 µM chenodeoxycholic acid (CDCA). In addition, newborn Sprague-Dawley rats were divided into the following groups: Control (n = 6), gavaged with vehicle alone once per day and Asbt Inhibitor (n = 6), gavaged with 5 ug/g/day SC-435 (Pfizer Inc., Groton, CT) once per day for 3 days. Ileal sections were removed and cultured as described above with and without 0.25 µM CDCA for 2.5 hours.

### Immunohistology

Tissue from explant studies was fixed in 70% ethanol, paraffin embedded and serial sectioned for immunohistologic evaluation. Tissue sections were evaluated for Muc2 and trefoil factor 3 (TFF3) positive cells using polyclonal, primary antibodies directed against Muc2 (Santa Cruz Biotechnology, Santa Cruz, CA) and TFF3 (kindly provided by Daniel K. Podolsky, UT Southwestern Medical Center, Dallas, TX). Positive cells were counted from at least 10 villi and data are expressed as mean positive cells per 100 intestinal epithelial cells.

### RNA preparation

Additional cultured explant sections were snap frozen and total RNA was isolated using the RNeasy Plus Mini Kit (Qiagen, Santa Clarita, CA) as described in the manufacturer's protocol. RNA concentration was quantified via ultraviolet spectrophotometry at 260 nm and its purity was determined via the A260/A280 ratio using a Nanodrop Spectrophotometer. Integrity of the RNA was verified by gel electrophoresis.

### Reverse Transcription (RT) and real-time PCR

Single-stranded cDNA was reverse-transcribed as previously described in detail [27]. The amounts of total RNA used in the RT reactions were calculated from the absorbency at 260 nm, and verified by densitometry of the 28S ribosomal RNA band separated on denaturing agarose gels (by Gel Doc 1000 Documentation System with Molecular Analyst/PC software, BIO-RAD, Hercules, CA). Real-time PCR amplification was performed using Assay By Design (Applied Biosystems, Foster City, CA) sequenced primers and probes for mouse IL-1β and TNF-α, and rat Muc2 and Muc3 (pre-made primer and probes). Samples were analyzed as previously described. [10], [22], [25].

### Immunoassay of secreted Muc2

Quantification of Muc2 secreted during ileal explant cultures was determined using an enzyme-linked immunosorbant assay (ELISA). The protocol described by Kim et. al, 2002 [28] was used except that supernatant from ileal explant cultures was incubated at 37°C in a 96-well ELISA plate until dry. Muc2 primary antibody (Santa Cruz Biotechnology) and a HRP-conjugated secondary antibody (Millipore, Billerica, MA) were utilized, color was developed with 3,3′, 5,5′-tetramethylbenzidine peroxidase and stopped after 20 mins with H_2_SO_4_. Absorbance was then read at 450 nm.

### Statistics

Statistical analyses between groups were performed using ANOVA followed by Fisher PLSD. The χ^2^ test was used to determine statistical differences in disease incidence. All numerical data are expressed as mean ± SD.

## Results

### Increased development of NEC in mice with aberrant Muc2

**Figure 1:**
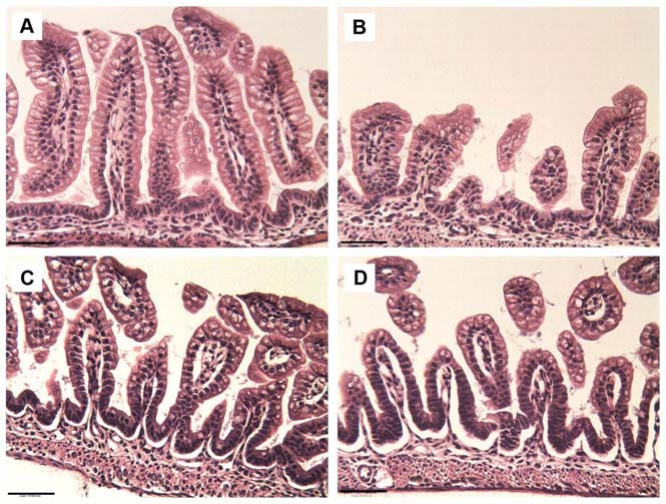
Increased ileal damage in mice with aberrant Muc2. Representative H & E staining of ileal sections from neonatal (A) Winnie −/− DF, (B) Winnie +/+ DF, (C) Winnie −/− NEC and (D) Winnie +/+ NEC mouse pups. Winnie −/− and +/+ DF depict ileal damage scores of 0; Winnie −/− NEC depicts damage score of 2, moderate separation of submucosa and/or lamina propria; Winnie +/+ NEC depicts damage score of 3, severe separation of submucosa and/or lamina propria. Bars indicate 50 µm.

**Figure 2:**
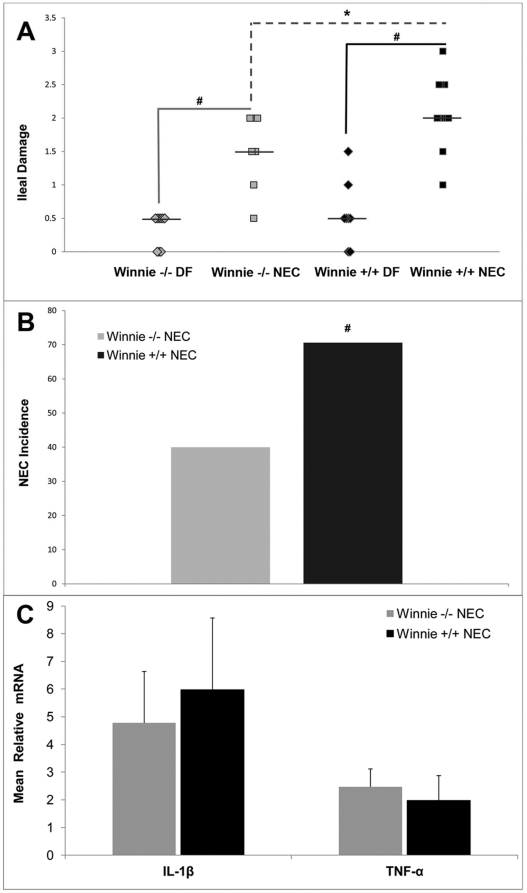
Increased severity and incidence of NEC in Winnie +/+ mice. A: Ileal damage score in Winnie −/− DF (n = 10), Winnie −/− NEC (n = 10), Winnie +/+ DF (n = 10) and Winnie +/+ NEC (n = 17) groups. Bars indicate median. B: Incidence of NEC in Winnie mice. Mice with damage scores ≥2 are considered to have NEC. C: Relative mRNA levels for IL-1β and TNF-α in Winnie −/− NEC and Winnie +/+ NEC groups. Mean steady-state mRNA for Winnie −/− DF and Winnie +/+ DF groups were assigned a value of 1.0 and the mean mRNA levels for the appropriate NEC group was determined relative to this number. Data are expressed as mean ± SD; n = 5 per group. * P≤0.05; # P≤0.01.

We have previously shown that Muc2 positive cells are significantly decreased in neonatal rats with NEC [15]. Winnie +/+ mice have fewer ileal goblet cells and a reduction of secreted mucus than wild-type littermates (Winnie −/−) [29]. To determine the role of Muc2 in NEC development, we subjected Winnie +/+ (Winnie +/+ NEC) and Winnie −/− (Winnie −/− NEC) mice to the NEC protocol (formula feeding with asphyxia/cold stress). As expected, DF Winnie −/− and +/+ mice did not develop NEC (Figure 1, Figure 2A). Interestingly, while the incidence of NEC for both DF strains was 0% (indicating no animals in either group has ileal damage score of 2 or greater), villi from Winnie +/+ DF were significantly shorter than those taken from Winnie −/− DF mice (81.6 µ±23.6 µ versus 122.7 µ±26.2 µ, respectively; P≤0.05). Winnie −/− NEC and +/+ NEC pups had significantly greater histological damage compared to their DF littermates (Figure 1 and Fig. 2A). However, Winnie +/+ NEC mice had significantly greater incidence and severity of NEC compared to Winnie −/− NEC mice (Figure 2A and 2B.). These data strongly suggest that deficiencies in Muc2 can exacerbate the development of experimental NEC. As adults, Winnie +/+ mice develop intestinal pathology with elevated IL-1β and TNF- α [29]. While both Winnie −/− and Winnie +/+ mice subjected to the NEC protocol had increased levels of pro-inflammatory cytokines compared to DF controls (4.8 and 5.9 fold, respectively), Winnie +/+ NEC mice did not have elevated IL-1β and TNF- α compared to Winnie −/− NEC mice (Figure 2C). Thus, the increased severity and incidence of NEC in the Winnie +/+ compared to Winnie −/− strain is not related to higher levels of pro-inflammatory cytokines.

### Removal of luminal BAs normalizes Muc2 positive cell decreases during NEC

**Figure 3:**
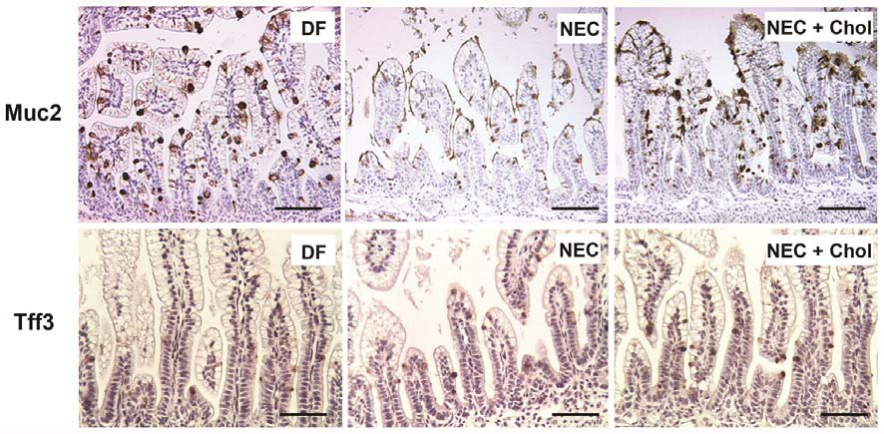
Muc2 positive cells are normalized in rats with NEC when BAs are pharmacologically sequestered with cholestyramine (Chol). Representative immunostaining of Muc2 (top panels) and Tff3 (bottom panels) positive cells in DF, animals subjected to the NEC protocol (NEC) and NEC animals given cholestyramine (NEC+Chol). Bars indicate 50 µm.

We have previously shown that luminal BAs are significantly elevated in both rats [10] and mice [11] with NEC with similar results in Winnie mice subjected to the NEC protocol (data not shown). Further, ileal Muc2 is decreased in NEC [15], and BAs are known to have effects on mucins [16]–[19]. To determine if the elevated luminal BAs observed in NEC [10] decrease ileal Muc2, neonatal rats subjected to the NEC protocol were given cholestyramine (Chol), a compound that binds BAs in the intestinal lumen. When rat pups subjected to the NEC protocol were given Chol (NEC+Chol), Muc2 mRNA levels were unchanged between groups (Table 1) however Muc2 positive cells were increased compared to NEC pups without Chol and were almost identical to those of DF pups (Figure 3, Table 1). Trefoil factors are goblet cell products that are co-secreted with mucins and play an important role in mucosal protection and repair [30]–[32]. Recently, we have shown that Tff3 positive cells are significantly elevated during the development of NEC in neonatal rats [33]. To determine if BAs affect goblet cell products universally, we determined the number of Tff3 positive cells in all experimental groups. Sequestration of BAs with Chol (NEC+Chol group) did not alter the increased Tff3 positive cells observed in the NEC group compared to DF pups (Figure 3, Table 1). Thus, pharmacologic sequestration of luminal BAs normalizes Muc2 positive but not Tff3 positive goblet cells during the development of experimental NEC.

**Table 1:** Muc2, Tff3 and luminal BA Levels in the Neonatal Rat NEC Model.

Group (n)	Muc2+cells^1^	Muc2 mRNA	Tff3+cells^1^	Total luminal BA^2^
**DF** (12)	7.5±0.3	1.0±0.1	3.3±0.3	0.7±0.2
**NEC** (12)	3.7±0.4*	1.2±0.3	5.6±0.7*	1.7±0.3#
**NEC+Chol** (12)	7.1±0.5	1.1±0.5	5.7±0.6*	0.3±0.1

1 Mean positive cells/100 intestinal epithelial cells ± SD.

2 Mean µg/L BAs/mg ileum ± SD.

Mean steady-state mRNA for the dam-fed (DF) group was assigned a value of 1.0 and the mean mRNA levels for the NEC and NEC+cholestyramine (NEC+Chol) groups were determined relative to this number.

*P≤0.05;

# *P≤0.01*.

### BAs decrease Muc2 positive cells in ex vivo ileal cultures from neonatal but not older rats

**Figure 4:**
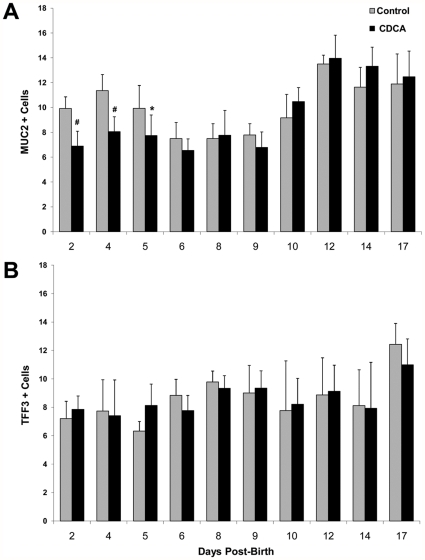
Muc2 and TFF3 in ileum from immature and more mature rats cultured with BAs. Ileal explants were cultured with 0.25 µM CDCA or media alone (control) for 2.5 hours. A: Muc2+cells and B: TFF3+cells in ileal explants from rats 2–17 days post-birth. Data are expressed as mean Muc2 or TFF3+cells/100 intestinal epithelial cells; n for each age = 8. ^#^ P≤0.01 versus control; * P≤0.05 versus control.

**Figure 5:**
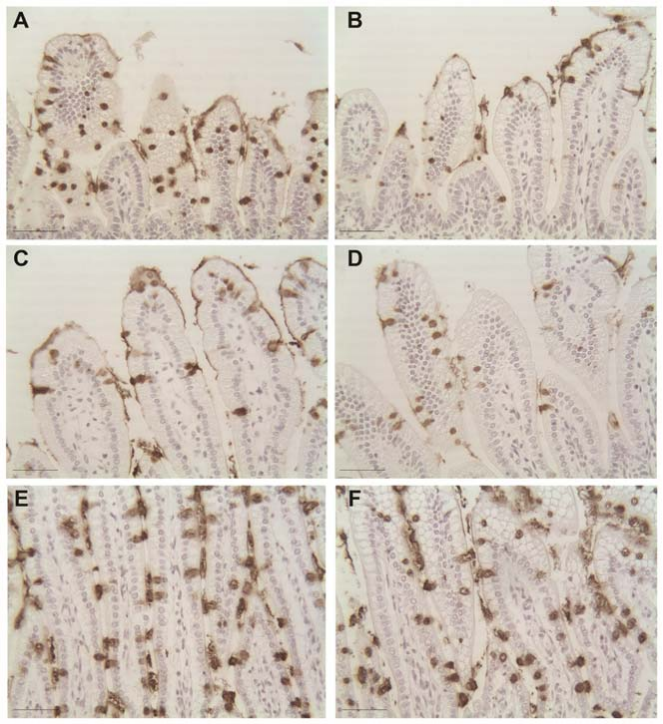
Muc2 staining of rat ileum cultured with CDCA. Representative ileal sections from a 3 day old rat cultured alone (A) or with CDCA (B), from a 6-day old rat cultured alone (C) or with CDCA (D) and a 17 day old rat cultured alone (E) or with CDCA (F). Bars indicate 50 µm.

**Figure 6:**
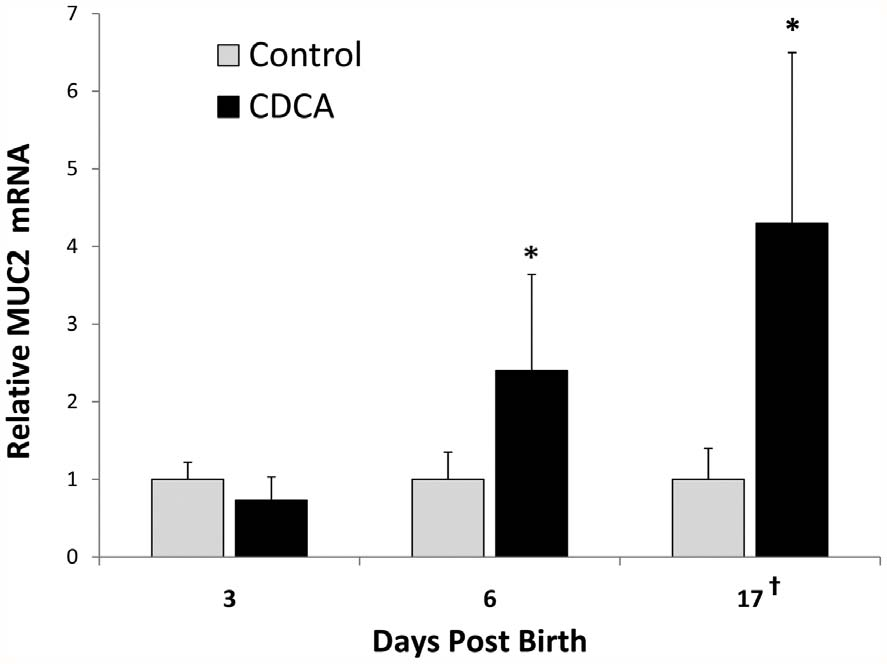
Relative Muc2 mRNA from CDCA cultured ileum from 3, 6 and 17 day old rats. Mean steady-state mRNA for each age-point's control group was assigned a value of 1.0 and the mean mRNA levels for the appropriate CDCA cultured group was determined relative to this number. Data are expressed as mean ± SD; n = 7 per group. **^†^** Mean values for 17 day old ileum are shown as a factor of 10. ^#^ P≤0.01 versus control;* P≤0.05 versus control.

Chol can bind ileal luminal compounds other than just BAs. To establish a direct role of BAs on Muc2, we utilized ileal explant cultures from neonatal rats. CDCA and deoxycholic acid (DCA) are the predominant BAs found in experimental NEC. We found that while 1.0, 0.5 and 0.25 µM CDCA reduced Muc2 positive cells, ileal tissue was damaged by the higher concentrations. In addition, DCA was extremely damaging to ileal cultured tissue at even lower concentrations tested (data not shown). Therefore, we chose to use 0.25 µM CDCA for all experiments. Muc2 positive cells were statistically significantly decreased in neonatal rat ileal explants cultured with CDCA only through day 5 post-birth. However, from day 6 to day 17, culture with CDCA did not affect the numbers of Muc2 positive cells (Figure 4A and Figure 5). Culture of rat ileal explants with CDCA had no effect on the number of Tff3 positive cells at any of the observed days post birth (Figure 4B). In addition, Muc2 mRNA levels were significantly unchanged in 3 day old rat ileum cultured with CDCA, but significantly increased in ileum taken from 6 and 17 day old rats (Figure 6). We have previously shown that during the development of NEC in neonatal rats, expression of Mucin 3 (Muc3), the primary non-secreted mucin in rat intestine, is significantly increased compared to DF littermates [33]. However, neonatal ileal explants exposed to CDCA showed no increase in Muc3 expression (data not shown). This strongly suggests that BAs have specific effects on Muc2 in neonatal ileum rather than on mucins in general.

### BAs induce Muc2 secretion in immature ileum

**Figure 7:**
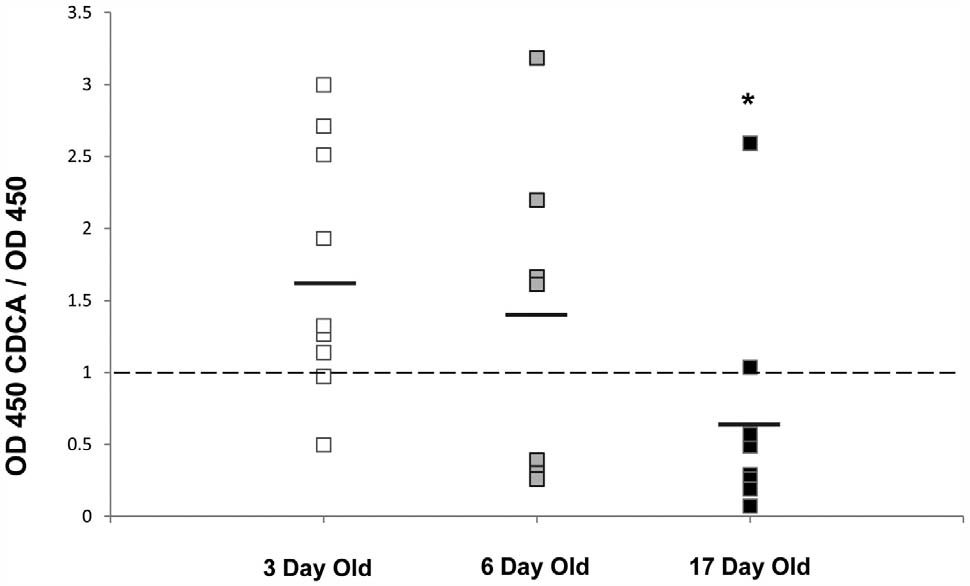
Secreted Muc2 in ileal explant cultures. Supernatants from 3 (n = 10), 6 (n = 7) and 17 day old (n = 10) rat ileal explants cultured with and without 0.25 µM CDCA for 2.5 hours were collected and assayed using ELISA. A: Data are shown as OD 450 for each animal's CDCA cultured ileal segment divided by OD 450 for the corresponding control ileal segment. * P≤0.05 versus 3 day old.

The decreased ileal Muc2 positive cells in 3 day old ileum after culture with CDCA could be a consequence of increased secretion of Muc2. Thus, supernatant from ileal explants from 3, 6 and 17 day old rats cultured with and without CDCA were analyzed via ELISA for Muc2 levels. 3 day old rat ileum cultured with CDCA secreted significantly more Muc2 compared to 17 day old ileal explants. There were no statistically significant differences between explants taken from 6 day old rats compared to either 3 or 17 day olds. (Figure 7).

### Immature ileum recovers its ability to produce Muc2

**Figure 8:**
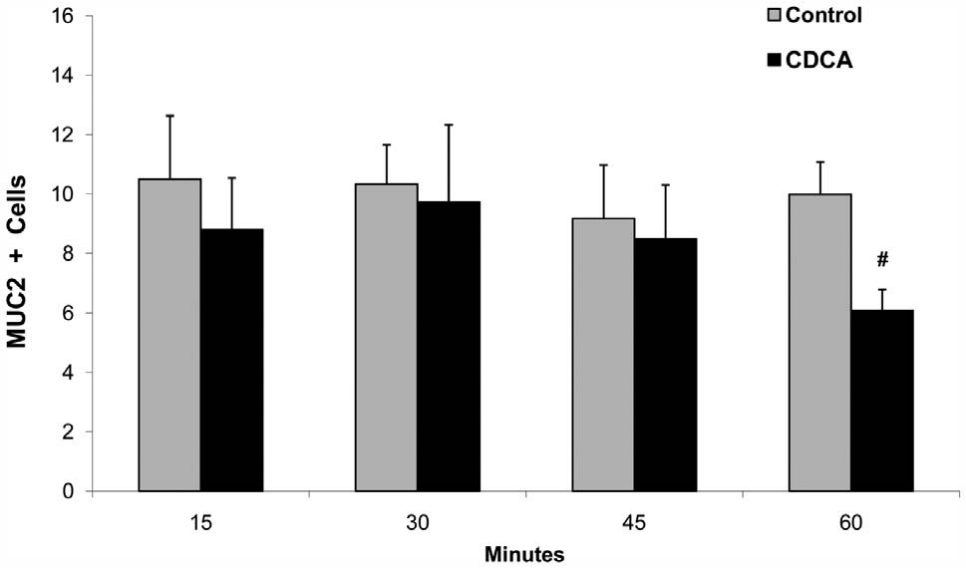
Temporal relationship between culture of ileal sections from 3 day old rats and Muc2+cells. Explants were cultured with 0.25 µM CDCA or media alone (control). Data are expressed as mean Muc2+cells/100 intestinal epithelial cells; n for each time point = 6. ^#^ P≤0.01 versus control.

The decrease of Muc2 positive cells in immature ileum did not occur immediately after culture with CDCA as no decreases were observed before 60 minutes (Figure 8). To determine if neonatal ileum is able to replenish Muc2 after exposure to BAs, we cultured ileum from 3 and 17 day old rats with CDCA for 2.5 hours, then washed and re-cultured the tissue in media alone for an additional 24 hours. Ileal tissue from 3 day old rats recovered the ability to produce Muc2 after 24 hours. Muc2 positive cells from older rats were significantly higher after 24 hours than after the initial culture, but there was no significant difference between control and CDCA treated tissue (Table 2).

**Table 2:** Recovery of Muc2+cells in rat ileum cultured with CDCA.

Days Post Birth	Control2.5 hr	CDCA2.5 hr	Control 24 hrRecovery	CDCA 24 hrRecovery
3	11.9±1.3	6.7±0.9*	12.6±3.8	16.9±4.1
17	12.7±2.0	13.3±3.1	20.4±5.2‡	22.5±6.1#

Ileum from 3 (n = 8) or 17 day old (n = 8) rats was cultured with or without CDCA for 2.5 hours, then washed and re-cultured in media alone for 24 hours. Data are expressed as mean Muc2+cells/100 intestinal epithelial cells ± SD.

*P≤0.05 versus 2.5 hr control;

‡ P≤0.01 versus 2.5 hr control;

# P≤0.01 versus 2.5 hr CDCA.

### Active transport of BAs is required for decreases in Muc2

**Figure 9:**
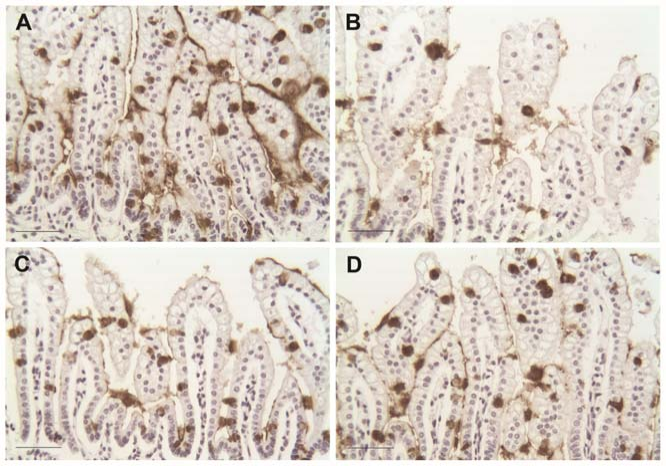
Muc2 staining of 3 day old WT and Asbt KO mouse ileal sections cultured with CDCA. WT mouse ileum was cultured alone (A) or with CDCA (B) and Asbt KO mouse ileum was cultured alone (C) or with CDCA (D). Bars indicate 50 µm.

Ileal luminal BAs are transported across the apical membrane of enterocytes via Asbt. To investigate if active transport of BAs is required to decrease Muc2 positive cells, newborn rats were given the Asbt inhibitor SC-435 [11] for 3 days before ileal sections were removed for explant studies. After inhibition of Asbt, there was no decrease in Muc2 positive cells in rat ileum cultured with CDCA (Table 3). Asbt knock-out mice (Asbt KO) cannot actively transport BAs across the apical membrane; therefore, these mice provide an additional model to examine if active transport of BAs is essential to the decrease in Muc2 positive cells in neonatal ileum. Similar to our results in immature rat ileum, Muc2 positive cells were decreased in CDCA-cultured ileal explants from 3 day old WT mice. However, there was no significant difference in Muc2 positive cells from CDCA-cultured Asbt KO ileal explants (Table 4 and Figure 9).

**Table 3:** Muc2 positive cells in CDCA-cultured, 3 day old rat ileum previously treated with an ASBT inhibitor.

Group	N	Muc2+Cells^1^
Vehicle Control	6	12.4±1.6
Vehicle CDCA	6	8.8±1.1*
ASBT Inhibitor Control	6	12.2±2.4
ASBT Inhibitor CDCA	6	13.5±1.9

1 Mean/100 intestinal epithelial cells ± SD.

Newborn rats were given vehicle alone or the ASBT inhibitor SC-435 for 3 days. Ileal explants were then taken and cultured with either media alone (control) or 0.25 µM CDCA for 2.5 hours.

*P≤0.05.

**Table 4:** Muc2+cells in CDCA cultured WT and ASBT KO mouse ileum.

Group	N	Muc2+Cells^1^
WT control	12	9.8±1.8
WT CDCA	12	7.2±1.5*
ASBT KO control	14	10.2±2.2
ASBT KO CDCA	14	10.7±2.3

1 Mean/100 intestinal epithelial cells ± SD.

Ileal explants from 3 day old wild-type (WT) and ASBT knock-out (ASBT KO) mice were cultured with 0.25 mM CDCA or media alone (control) for 2.5 hours.

*P≤0.05.

**Figure 10:**
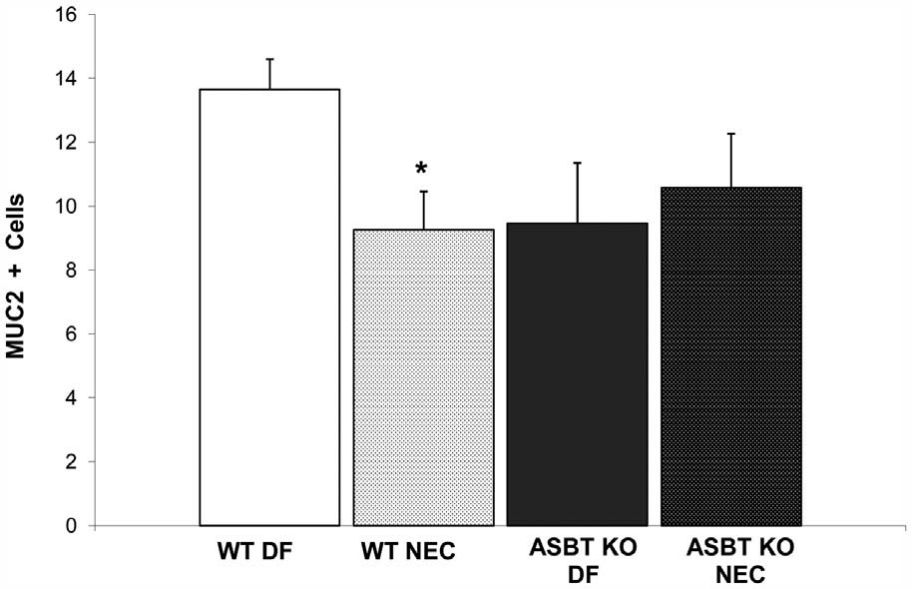
Muc2+cells in wild-type dam-fed (WT DF), wild-type mice subjected to the NEC protocol (WT NEC), Asbt knock-out dam-fed (Asbt KO DF) and Asbt KO mice subjected to the NEC protocol (Asbt KO NEC). Data are expressed as mean Muc2+cells/100 intestinal epithelial cells; n = 10 per each experimental group * P≤0.05 versus WT DF.

When subjected to the NEC protocol, Asbt KO mice have elevated luminal BAs, significantly diminished intra-enterocyte BA levels and develop significantly less disease compared to WT mice [11]. As expected, WT mice subjected to the NEC protocol (WT NEC) had significantly less Muc2 positive cells compared to WT DF mice. However, there was no difference between Muc2 positive cells between Asbt KO DF and Asbt KO NEC mice (Figure 10). These data indicate that active transport of BAs via Asbt is essential for the reduction of Muc2 positive cells in immature ileum.

## Discussion

Accumulation of ileal BAs is known to play a role in intestinal pathology and inflammation [34]–[36]. Further, we have previously shown that BAs play a significant role in the development of experimental NEC [10] and the up-regulation of Asbt is essential for disease occurrence [11]. We have also shown that Muc2, the predominant secreted mucin in rodents, is significantly reduced during the development of experimental NEC [15], [33]. In the studies presented herein, we show that Muc2 plays an important role in the pathogenesis of NEC and BAs selectively decrease Muc2 in neonatal but not older ileum. In addition, active transport of BAs across the apical membrane of enterocytes is required for BAs to decrease Muc2 in neonatal ileum. These data suggest that BAs have a more profound effect on Muc2 from neonatal compared to more mature ileum, which has implications for the pathophysiology of NEC, a disease that occurs almost exclusively in premature infants.

Mucins protect the epithelial surface of the GI tract by forming a mucous layer between the intestinal lumen and epithelium [12], [13], [37]. We have previously shown that the goblet cell product Muc2, but not Tff3, is significantly decreased during the development of experimental NEC [15]. Here, we show that mice with aberrant Muc2 are more susceptible to NEC, strongly implicating the role of Muc2 in disease development. BAs have been shown to alter Muc2 secretion [16]–[19] and mucus layer deficiency has been suggested to contribute to intestinal injury during NEC [14]. In the studies presented herein, we show that BAs play a crucial role in formation of the mucus layer in experimental NEC as reduction of ileal BAs significantly increases Muc2 positive cells in rats subjected to the NEC protocol. Further, *ex vivo* studies showed that while BAs decreased Muc2 positive cells in neonatal ileal explants, not all goblet cell products were similarly affected as Tff3 positive cells were not decreased after exposure to BAs. Thus, accumulating ileal BAs are a likely mediator of the decreases in Muc2 observed in NEC.

We also found that less mature rat ileum reacts differently to treatment with BAs than does more mature ileum. Up to 6 days post birth, treatment of ileal explants with CDCA results in a statistically significant decrease in Muc2 positive cells; from 6 to 17 days, no noticeable change in the number of Muc2 positive cells was observed. Interestingly, a recent publication by McElroy et al. showed developmental differences in the effects of TNF-α on Muc2 [38]. Why BAs decrease Muc2 positive cells only in neonatal ileum is not yet understood, but there are a number of possible explanations for the differences. There are significant physical and chemical differences in neonatal versus adult intestinal mucus glycoproteins [39] that could influence how BAs effect mucins in immature and mature intestine. It is unclear, however, at what age these differences—lower fucose content, lower buoyant density in CsCl and fewer carbohydrate side chains in newborn rat mucus—change to an adult profile. It is more likely that these characteristics are affected at the time of weaning and not as early as day 6 post birth, however. BAs have also been shown to regulate Muc2 transcription via NF-κB, AP-1 and the EGF receptor [40]–[42], pathways that are developmentally regulated [43]–[45]. Studies to examine these transcription factors in BA cultured ileum from immature and mature rats are currently underway in our laboratory.

Previous work has shown that BAs can stimulate mucin secretion in a human colon cell line [17] and up-regulate Muc2 in human esophageal [46] and colon carcinoma cells. Three day old ileum exposed to BA secrete significantly more Muc2 compared to control than do 17 day old ileum and these data suggest that the decreased Muc2 positive cells in immature ileum after BA treatment are a result of increased secretion of Muc2. Immature ileum was capable of replenishing Muc2 after initial secretion, but had significantly lower levels of Muc2 compared to more mature ileum after the recovery period. Thus, the immature ileum may be less able to protect itself from exposure to BAs compared to more mature ileum.

BAs have detergent properties that can have toxic effects on cells [47], [48]. These properties could elicit damage to the intestinal epithelium and lead to secretion of Muc2 as a protective mechanism. However, Klinspoor et al, showed that bile salts can stimulate mucin secretion in dog gallbladder epithelium independent of their detergent effect [49]. In this study, Muc2 positive cells were not decreased in CDCA-cultured Asbt-inhibited neonatal rat and Asbt knock-out mouse ileum. These data strongly suggest that the ability of BAs to decrease Muc2 positive cells in neonatal ileum requires an active process of BA transport into enterocytes, and not simply a consequence of physical contact with the intestinal epithelium. Asbt expression in rats is high prior to birth, but is significantly suppressed postnatally until it is again up-regulated at the time of weaning [50]. In both experimental and human NEC, Asbt is increased [11] and elevated intra-enterocyte BA levels are associated with incidence and severity of experimental NEC [10]. These data suggest that increased ileal BA levels coupled with up-regulation of Asbt contribute to the development of NEC because the mucin layer in immature ileum is more sensitive to the effects of BA accumulation that older ileum.

In summary, Muc2 plays a crucial role in the development of NEC and elevated ileal BAs likely contribute to decreased Muc2 positive cells seen in this disease. Further, active transport of BAs is required for this decrease in Muc2 positive cells. Importantly, the inability to increase Muc2 in response to elevated BA levels in immature ileum may explain, in part, why NEC occurs almost exclusively in premature infants. These data could influence the development of future therapeutic modalities to decrease ileal BA levels in premature infants at high risk to develop NEC.
